# [μ_2_-*N* ^2^,*N* ^2′^-Bis(3-meth­oxy-2-oxido­benzyl­idene)benzene-1,3-dicarbo­hydrazi­dato]bis­[pyridine­copper(II)]

**DOI:** 10.1107/S1600536813030286

**Published:** 2013-11-13

**Authors:** Hai-Bin Tong, Zi-Jing Xiao

**Affiliations:** a College of Materials Science and Engineering, Huaqiao University, Xiamen, Fujian 361021, People’s Republic of China

## Abstract

In the centrosymmetric dinuclear title complex, [Cu_2_(C_24_H_18_N_4_O_6_)(C_5_H_5_N)_2_], the Cu^II^ ions is tetra­coordinated by two O-atoms and one N-donor of the bridging terephthalo­hydra­zonate ligand and by one pyridine N atom, resulting in a nearly square-planar N_2_O_2_ coordination geometry with the Cu^II^ ion 0.044 (2) Å out of the mean plane (r.m.s. deviation of 0.0675 Å) of the coordinating atoms.

## Related literature

For the structural coordination chemistry and potential applications in luminescence, redox activity and magnetism of bifunctional organic ligands and their complexes, see: He *et al.* (2004 ▶); Qiao *et al.* (2007 ▶); Yin *et al.* (2008 ▶); Zhu *et al.* (2010 ▶); Lin *et al.* (2012 ▶). For the crystal structures of dinuclear copper(II) complexes with a similar coordination geometry, see: Banerjee *et al.* (2009 ▶); Shulgin *et al.* (2011 ▶); Mistri *et al.* (2013 ▶). For the synthesis of *N*,*N′*-bis­(3-meth­oxy-2-oxybenzyl­idene)terephthalohydrazone, see: Yin *et al.* (2008 ▶).
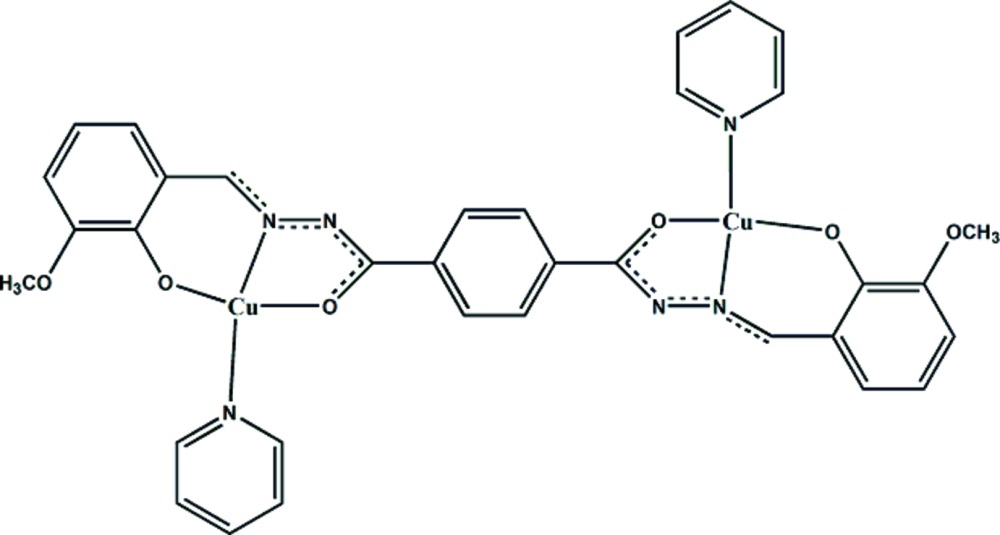


## Experimental

### 

#### Crystal data

[Cu_2_(C_24_H_18_N_4_O_6_)(C_5_H_5_N)_2_]
*M* *_r_* = 743.70
Monoclinic, 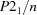

*a* = 4.8474 (2) Å
*b* = 15.2776 (6) Å
*c* = 20.5546 (6) Å
β = 96.113 (4)°
*V* = 1513.55 (10) Å^3^
*Z* = 2
Cu *K*α radiation
μ = 2.23 mm^−1^
*T* = 153 K
0.45 × 0.32 × 0.22 mm

#### Data collection

Agilent Gemini S Ultra diffractometer
Absorption correction: multi-scan (*CrysAlis PRO*; Agilent, 2012 ▶) *T*_min_ = 0.447, *T*_max_ = 0.615
5837 measured reflections
2658 independent reflections
2113 reflections with *I* > 2σ(*I*)
*R*_int_ = 0.029

#### Refinement

*R*[*F* ^2^ > 2σ(*F* ^2^)] = 0.045
*wR*(*F* ^2^) = 0.133
*S* = 1.06
2658 reflections
218 parameters
H-atom parameters constrained
Δρ_max_ = 0.38 e Å^−3^
Δρ_min_ = −0.35 e Å^−3^

### 

Data collection: *CrysAlis PRO* (Agilent, 2012 ▶); cell refinement: *CrysAlis PRO*; data reduction: *CrysAlis PRO*; program(s) used to solve structure: *SHELXS97* (Sheldrick, 2008 ▶); program(s) used to refine structure: *SHELXL97* (Sheldrick, 2008 ▶); molecular graphics: *DIAMOND* (Brandenburg, 2006 ▶); software used to prepare material for publication: *publCIF* (Westrip, 2010 ▶).

## Supplementary Material

Crystal structure: contains datablock(s) I, a. DOI: 10.1107/S1600536813030286/fj2648sup1.cif

Structure factors: contains datablock(s) I. DOI: 10.1107/S1600536813030286/fj2648Isup2.hkl

Additional supplementary materials:  crystallographic information; 3D view; checkCIF report

## Figures and Tables

**Fig. 1.**
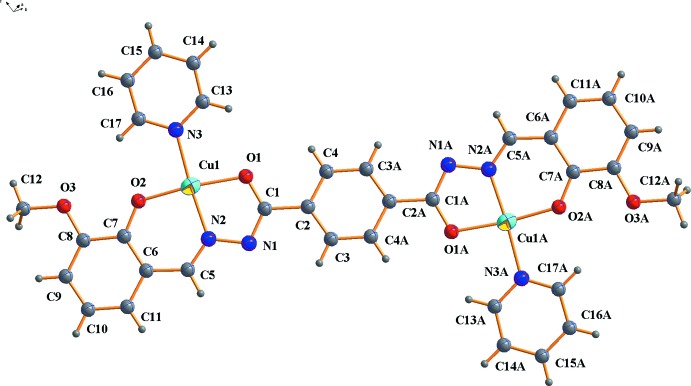
The molecular structure of the title complex, showing 50% probability displacement ellipsoids and the atom numbering scheme.

## supplementary crystallographic information

### 1. Comment

Bifunctional organic ligand and their complexes have attracted increasing interest in recent years, due to their potential applications in luminescence, redox activity, magnetism and diversities of coordination (He *et al.*, 2004; Qiao *et al.*, 2007; Yin *et al.*, 2008; Zhu *et al.*, 2010; Lin *et al.*, 2012). Relatively speaking, only a few crystal structures of arylaldehyde terephthalohydrazone complexes have been reported (He *et al.*, 2004; Yin *et al.*,2008; Lin *et al.*, 2012). Herein, we report the synthesis and structure of copper complex with *N*,*N*'-bis(3-methoxy-2-oxybenzylidene)terephthalohydrazone.

As show in Fig 1, we can see that the title complex contain dinuclear copper(II) skeletons showing a *trans* conformation (centrosymmetry). That is, one half of the dinuclear copper(II) complex constitutes the crystallographic asymmetric unit and the other half is produced by an inversion centre. In the title complex, Cu1(II) ion is coordinated by carbonyl atom O1, hydrazine atom N2 and phenol atom O2 from the moieties of the ligand H_4_L, which is hexadentate ligand that function as tetrabasic in the enol form, and N3 atom from coordinated pyridine molecule, obtaining a nearly square-planar N_2_O_2_ geometry (r.m.s deviation =0.0675 Å). The Cu^II^ atom is shifted 0.044 (2) Å out of the square-plane. One five-membered chelate ring (ring *M*_1_) and one six-membered chelate ring (ring *M*_2_) are formed by the moieties of the ligand *L*^4-^ and Cu1 atom. The ring *M*_1_ is composed of Cu1, N2, N1, C1 and O1 with r.m.s deviation of 0.0123 Å,The ring *M*_2_ of Cu1, N2, C5, C6, C7 and O2 with r.m.s deviation of 0.0238 Å. All two chelate rings are planar. The bond lengths of Cu—N and Cu—O in the title complex are similar to those in other dinuclear copper(II) complexes (Banerjee *et al.*, 2009; Shulgin *et al.*, 2011; Mistri *et al.*, 2013). The whole *L*^4-^ ligand is a nearly planar (r.m.s deviation=0.0805 Å), the dihedral angle between the two benzene rings is 7.29 (29)°.

### 2. Experimental

Reagents and solvents were used as obtained without further purification. *N*,*N*'-bis(3-methoxy-2-oxybenzylidene)terephthalohydrazone (H_4_L) was synthesized according to the literature methods (Yin *et al.*, 2008). H_4_L (0.0462 g, 0.1 mmol) and copper(II) chloride dihydrate (0.0342 g, 0.2 mmol) were dissolved in a mixed solution of 10 ml CH_3_OH and 5 ml DMF. The mixture was stirred for 10 minutes at room temperature and then 5 ml pyridine was slowly added. The reaction mixture was further stirred for 4 h. After being obtained by filtration, the dark green filtrate was allowed to stand at room temperature for 15 days. The dark green prism crystals of the title complex were obtained by slow evaporation.

### 3. Refinement

All H atoms were positioned geometrically and refined using a riding model [C—H=0.93 Å and *U*_iso_(H) = 1.2Ueq(C) for aromatic H atoms,*C*—H=0.96 Å and *U*_iso_(H) = 1.5Ueq(*c*) for methyl H atoms].

### Crystal data

**Table:**

[Cu_2_(C_24_H_18_N_4_O_6_)(C_5_H_5_N)_2_]	*F*(000) = 760
*M**_r_* = 743.70	*D*_x_ = 1.632 Mg m^−^^3^
Monoclinic, *P*2_1_/*n*	Cu *K*α radiation, λ = 1.54178 Å
Hall symbol: -P 2yn	Cell parameters from 2658 reflections
*a* = 4.8474 (2) Å	θ = 3.6–66.5°
*b* = 15.2776 (6) Å	µ = 2.23 mm^−^^1^
*c* = 20.5546 (6) Å	*T* = 153 K
β = 96.113 (4)°	Prism, dark green
*V* = 1513.55 (10) Å^3^	0.45 × 0.32 × 0.22 mm
*Z* = 2	

### Data collection

**Table:**

Agilent Gemini S Ultra diffractometer	2658 independent reflections
Radiation source: Enhance Ultra (Cu) X-ray Source	2113 reflections with *I* > 2σ(*I*)
Mirror monochromator	*R*_int_ = 0.029
Detector resolution: 15.9149 pixels mm^-1^	θ_max_ = 66.5°, θ_min_ = 3.6°
ω scans	*h* = −3→5
Absorption correction: multi-scan (*CrysAlis PRO*; Agilent, 2012)	*k* = −17→17
*T*_min_ = 0.447, *T*_max_ = 0.615	*l* = −24→24
5837 measured reflections	

### Refinement

**Table:**

Refinement on *F*^2^	Primary atom site location: structure-invariant direct methods
Least-squares matrix: full	Secondary atom site location: difference Fourier map
*R*[*F*^2^ > 2σ(*F*^2^)] = 0.045	Hydrogen site location: inferred from neighbouring sites
*wR*(*F*^2^) = 0.133	H-atom parameters constrained
*S* = 1.06	*w* = 1/[σ^2^(*F*_o_^2^) + (0.0637*P*)^2^ + 0.5957*P*] where *P* = (*F*_o_^2^ + 2*F*_c_^2^)/3
2658 reflections	(Δ/σ)_max_ < 0.001
218 parameters	Δρ_max_ = 0.38 e Å^−^^3^
0 restraints	Δρ_min_ = −0.35 e Å^−^^3^

### Special details

**Table:**

Experimental. *CrysAlis PRO*, Agilent Technologies, Version 1.171.36.21 (release 14–08-2012 CrysAlis171. NET) (compiled Sep 14 2012,17:21:16) Empirical absorption correction using spherical harmonics, implemented in SCALE3 ABSPACK scaling algorithm.
Geometry. All e.s.d.'s (except the e.s.d. in the dihedral angle between two l.s. planes) are estimated using the full covariance matrix. The cell e.s.d.'s are taken into account individually in the estimation of e.s.d.'s in distances, angles and torsion angles; correlations between e.s.d.'s in cell parameters are only used when they are defined by crystal symmetry. An approximate (isotropic) treatment of cell e.s.d.'s is used for estimating e.s.d.'s involving l.s. planes.
Refinement. Refinement of *F*^2^ against ALL reflections. The weighted *R*-factor *wR* and goodness of fit *S* are based on *F*^2^, conventional *R*-factors *R* are based on *F*, with *F* set to zero for negative *F*^2^. The threshold expression of *F*^2^ > σ(*F*^2^) is used only for calculating *R*-factors(gt) *etc*. and is not relevant to the choice of reflections for refinement. *R*-factors based on *F*^2^ are statistically about twice as large as those based on *F*, and *R*- factors based on ALL data will be even larger.

### Fractional atomic coordinates and isotropic or equivalent isotropic displacement parameters (Å^2^)

**Table:**

	*x*	*y*	*z*	*U*_iso_*/*U*_eq_	
Cu1	0.10363 (10)	0.25720 (3)	0.16238 (2)	0.0517 (2)	
N1	0.0548 (6)	0.3121 (2)	0.03092 (13)	0.0562 (7)	
N2	−0.0561 (6)	0.25260 (18)	0.07303 (14)	0.0508 (7)	
N3	0.2965 (6)	0.26684 (19)	0.25349 (14)	0.0511 (7)	
O1	0.3097 (5)	0.35162 (17)	0.12788 (10)	0.0565 (6)	
O2	−0.1445 (5)	0.17419 (17)	0.19006 (11)	0.0602 (6)	
O3	−0.4238 (6)	0.0629 (2)	0.25128 (12)	0.0706 (8)	
C1	0.2389 (7)	0.3600 (2)	0.06565 (16)	0.0511 (8)	
C2	0.3731 (7)	0.4314 (2)	0.03120 (16)	0.0528 (8)	
C3	0.3252 (9)	0.4435 (3)	−0.03547 (17)	0.0730 (12)	
H3A	0.2067	0.4054	−0.0603	0.088*	
C4	0.5515 (9)	0.4894 (3)	0.06609 (18)	0.0733 (12)	
H4A	0.5884	0.4828	0.1112	0.088*	
C5	−0.2521 (8)	0.2022 (3)	0.04869 (17)	0.0578 (9)	
H5A	−0.3074	0.2070	0.0041	0.069*	
C6	−0.3922 (7)	0.1392 (2)	0.08483 (17)	0.0541 (8)	
C7	−0.3333 (7)	0.1298 (2)	0.15349 (17)	0.0530 (8)	
C8	−0.4884 (8)	0.0671 (3)	0.18484 (18)	0.0564 (9)	
C9	−0.6886 (8)	0.0168 (3)	0.1499 (2)	0.0666 (10)	
H9A	−0.7900	−0.0235	0.1715	0.080*	
C10	−0.7394 (8)	0.0262 (3)	0.0824 (2)	0.0684 (11)	
H10A	−0.8718	−0.0088	0.0589	0.082*	
C11	−0.5966 (8)	0.0862 (3)	0.05070 (19)	0.0643 (10)	
H11A	−0.6343	0.0924	0.0056	0.077*	
C12	−0.5910 (10)	0.0070 (3)	0.2862 (2)	0.0793 (13)	
H12A	−0.5422	0.0145	0.3324	0.119*	
H12B	−0.5606	−0.0528	0.2745	0.119*	
H12C	−0.7830	0.0216	0.2753	0.119*	
C13	0.5123 (8)	0.3210 (2)	0.26756 (17)	0.0571 (9)	
H13A	0.5736	0.3542	0.2340	0.069*	
C14	0.6461 (8)	0.3293 (3)	0.32932 (19)	0.0648 (10)	
H14A	0.7951	0.3675	0.3373	0.078*	
C15	0.5587 (9)	0.2807 (3)	0.37955 (19)	0.0665 (10)	
H15A	0.6463	0.2857	0.4219	0.080*	
C16	0.3409 (9)	0.2250 (3)	0.36585 (18)	0.0651 (10)	
H16A	0.2792	0.1908	0.3988	0.078*	
C17	0.2124 (8)	0.2198 (3)	0.30282 (17)	0.0583 (9)	
H17A	0.0619	0.1823	0.2942	0.070*	

### Atomic displacement parameters (Å^2^)

**Table:**

	*U*^11^	*U*^22^	*U*^33^	*U*^12^	*U*^13^	*U*^23^
Cu1	0.0608 (3)	0.0568 (3)	0.0367 (3)	−0.0049 (2)	0.0017 (2)	0.0011 (2)
N1	0.0682 (18)	0.0619 (18)	0.0379 (14)	−0.0119 (15)	0.0030 (13)	0.0058 (14)
N2	0.0598 (16)	0.0557 (17)	0.0365 (14)	−0.0046 (14)	0.0035 (12)	0.0003 (12)
N3	0.0583 (16)	0.0568 (17)	0.0377 (14)	0.0027 (14)	0.0026 (12)	0.0013 (13)
O1	0.0745 (15)	0.0598 (14)	0.0348 (11)	−0.0132 (12)	0.0035 (10)	0.0020 (10)
O2	0.0668 (15)	0.0699 (16)	0.0429 (12)	−0.0143 (13)	0.0011 (11)	0.0015 (12)
O3	0.0825 (18)	0.0779 (18)	0.0502 (14)	−0.0214 (15)	0.0014 (13)	0.0066 (13)
C1	0.061 (2)	0.0542 (19)	0.0382 (16)	0.0007 (16)	0.0072 (14)	0.0010 (15)
C2	0.064 (2)	0.0564 (19)	0.0377 (16)	−0.0036 (17)	0.0056 (14)	0.0031 (15)
C3	0.093 (3)	0.084 (3)	0.0399 (18)	−0.037 (2)	−0.0064 (18)	0.0040 (19)
C4	0.098 (3)	0.084 (3)	0.0352 (17)	−0.032 (2)	−0.0025 (18)	0.0083 (19)
C5	0.065 (2)	0.069 (2)	0.0384 (17)	−0.0003 (19)	0.0012 (15)	0.0005 (17)
C6	0.0566 (19)	0.056 (2)	0.0488 (19)	0.0008 (16)	0.0015 (15)	−0.0029 (16)
C7	0.0555 (18)	0.055 (2)	0.0479 (18)	−0.0001 (16)	0.0034 (15)	−0.0024 (16)
C8	0.061 (2)	0.055 (2)	0.053 (2)	−0.0017 (17)	0.0044 (16)	−0.0002 (17)
C9	0.063 (2)	0.066 (2)	0.070 (3)	−0.0103 (19)	0.0030 (19)	0.003 (2)
C10	0.066 (2)	0.069 (2)	0.067 (3)	−0.013 (2)	−0.0083 (19)	−0.004 (2)
C11	0.067 (2)	0.068 (2)	0.054 (2)	−0.004 (2)	−0.0074 (17)	−0.0025 (19)
C12	0.099 (3)	0.077 (3)	0.061 (2)	−0.025 (3)	0.008 (2)	0.011 (2)
C13	0.062 (2)	0.059 (2)	0.0490 (19)	−0.0002 (18)	0.0022 (16)	0.0064 (17)
C14	0.062 (2)	0.068 (2)	0.062 (2)	−0.0012 (19)	−0.0088 (18)	−0.005 (2)
C15	0.074 (2)	0.080 (3)	0.0429 (19)	0.009 (2)	−0.0090 (17)	−0.0009 (19)
C16	0.080 (3)	0.073 (3)	0.0418 (19)	0.001 (2)	0.0011 (18)	0.0078 (18)
C17	0.067 (2)	0.063 (2)	0.0446 (19)	−0.0036 (18)	0.0009 (16)	0.0065 (17)

### Geometric parameters (Å, º)

**Table:**

Cu1—O2	1.877 (3)	C6—C11	1.407 (5)
Cu1—N2	1.917 (3)	C6—C7	1.417 (5)
Cu1—O1	1.932 (2)	C7—C8	1.414 (5)
Cu1—N3	2.007 (3)	C8—C9	1.379 (5)
N1—C1	1.307 (5)	C9—C10	1.389 (5)
N1—N2	1.401 (4)	C9—H9A	0.9300
N2—C5	1.282 (5)	C10—C11	1.357 (5)
N3—C13	1.341 (5)	C10—H10A	0.9300
N3—C17	1.341 (5)	C11—H11A	0.9300
O1—C1	1.295 (4)	C12—H12A	0.9600
O2—C7	1.310 (4)	C12—H12B	0.9600
O3—C8	1.370 (4)	C12—H12C	0.9600
O3—C12	1.423 (4)	C13—C14	1.368 (5)
C1—C2	1.487 (5)	C13—H13A	0.9300
C2—C3	1.378 (5)	C14—C15	1.375 (6)
C2—C4	1.384 (5)	C14—H14A	0.9300
C3—C4^i^	1.373 (5)	C15—C16	1.361 (6)
C3—H3A	0.9300	C15—H15A	0.9300
C4—C3^i^	1.373 (5)	C16—C17	1.379 (5)
C4—H4A	0.9300	C16—H16A	0.9300
C5—C6	1.431 (5)	C17—H17A	0.9300
C5—H5A	0.9300		
			
O2—Cu1—N2	93.38 (11)	O2—C7—C6	125.0 (3)
O2—Cu1—O1	171.34 (11)	C8—C7—C6	117.5 (3)
N2—Cu1—O1	81.23 (11)	O3—C8—C9	124.6 (4)
O2—Cu1—N3	90.95 (11)	O3—C8—C7	114.2 (3)
N2—Cu1—N3	175.52 (12)	C9—C8—C7	121.2 (3)
O1—Cu1—N3	94.59 (11)	C8—C9—C10	120.2 (4)
C1—N1—N2	108.1 (3)	C8—C9—H9A	119.9
C5—N2—N1	117.7 (3)	C10—C9—H9A	119.9
C5—N2—Cu1	127.1 (3)	C11—C10—C9	120.3 (4)
N1—N2—Cu1	115.2 (2)	C11—C10—H10A	119.9
C13—N3—C17	117.4 (3)	C9—C10—H10A	119.9
C13—N3—Cu1	121.4 (2)	C10—C11—C6	121.1 (4)
C17—N3—Cu1	121.2 (3)	C10—C11—H11A	119.4
C1—O1—Cu1	110.1 (2)	C6—C11—H11A	119.4
C7—O2—Cu1	127.4 (2)	O3—C12—H12A	109.5
C8—O3—C12	116.7 (3)	O3—C12—H12B	109.5
O1—C1—N1	125.3 (3)	H12A—C12—H12B	109.5
O1—C1—C2	117.4 (3)	O3—C12—H12C	109.5
N1—C1—C2	117.3 (3)	H12A—C12—H12C	109.5
C3—C2—C4	117.3 (3)	H12B—C12—H12C	109.5
C3—C2—C1	122.4 (3)	N3—C13—C14	122.6 (4)
C4—C2—C1	120.2 (3)	N3—C13—H13A	118.7
C4^i^—C3—C2	121.4 (4)	C14—C13—H13A	118.7
C4^i^—C3—H3A	119.3	C13—C14—C15	119.5 (4)
C2—C3—H3A	119.3	C13—C14—H14A	120.2
C3^i^—C4—C2	121.3 (3)	C15—C14—H14A	120.2
C3^i^—C4—H4A	119.4	C16—C15—C14	118.4 (4)
C2—C4—H4A	119.4	C16—C15—H15A	120.8
N2—C5—C6	125.1 (3)	C14—C15—H15A	120.8
N2—C5—H5A	117.5	C15—C16—C17	119.6 (4)
C6—C5—H5A	117.5	C15—C16—H16A	120.2
C11—C6—C7	119.6 (4)	C17—C16—H16A	120.2
C11—C6—C5	118.4 (3)	N3—C17—C16	122.4 (4)
C7—C6—C5	121.9 (3)	N3—C17—H17A	118.8
O2—C7—C8	117.5 (3)	C16—C17—H17A	118.8
			
C1—N1—N2—C5	176.5 (3)	N2—C5—C6—C11	−177.6 (4)
C1—N1—N2—Cu1	−2.8 (4)	N2—C5—C6—C7	3.2 (6)
O2—Cu1—N2—C5	−3.7 (3)	Cu1—O2—C7—C8	176.9 (3)
O1—Cu1—N2—C5	−176.9 (3)	Cu1—O2—C7—C6	−2.6 (5)
O2—Cu1—N2—N1	175.5 (2)	C11—C6—C7—O2	178.7 (4)
O1—Cu1—N2—N1	2.4 (2)	C5—C6—C7—O2	−2.2 (6)
O2—Cu1—N3—C13	−179.7 (3)	C11—C6—C7—C8	−0.8 (5)
O1—Cu1—N3—C13	−6.4 (3)	C5—C6—C7—C8	178.4 (3)
O2—Cu1—N3—C17	−0.3 (3)	C12—O3—C8—C9	−4.8 (6)
O1—Cu1—N3—C17	173.0 (3)	C12—O3—C8—C7	174.2 (3)
N2—Cu1—O1—C1	−1.4 (2)	O2—C7—C8—O3	1.7 (5)
N3—Cu1—O1—C1	177.0 (2)	C6—C7—C8—O3	−178.8 (3)
N2—Cu1—O2—C7	4.7 (3)	O2—C7—C8—C9	−179.2 (4)
N3—Cu1—O2—C7	−174.2 (3)	C6—C7—C8—C9	0.3 (6)
Cu1—O1—C1—N1	0.1 (5)	O3—C8—C9—C10	179.8 (4)
Cu1—O1—C1—C2	178.6 (2)	C7—C8—C9—C10	0.8 (6)
N2—N1—C1—O1	1.8 (5)	C8—C9—C10—C11	−1.4 (6)
N2—N1—C1—C2	−176.8 (3)	C9—C10—C11—C6	0.9 (6)
O1—C1—C2—C3	176.4 (4)	C7—C6—C11—C10	0.2 (6)
N1—C1—C2—C3	−4.9 (6)	C5—C6—C11—C10	−179.0 (4)
O1—C1—C2—C4	−4.3 (5)	C17—N3—C13—C14	0.0 (6)
N1—C1—C2—C4	174.3 (4)	Cu1—N3—C13—C14	179.5 (3)
C4—C2—C3—C4^i^	−0.2 (8)	N3—C13—C14—C15	0.1 (6)
C1—C2—C3—C4^i^	179.1 (4)	C13—C14—C15—C16	0.4 (6)
C3—C2—C4—C3^i^	0.2 (8)	C14—C15—C16—C17	−0.9 (6)
C1—C2—C4—C3^i^	−179.1 (4)	C13—N3—C17—C16	−0.6 (6)
N1—N2—C5—C6	−178.7 (3)	Cu1—N3—C17—C16	180.0 (3)
Cu1—N2—C5—C6	0.6 (6)	C15—C16—C17—N3	1.0 (6)

Symmetry code: (i) −*x*+1, −*y*+1, −*z*.
